# The effect of preoperative hypoalbuminemia on complications after primary hip arthroplasty

**DOI:** 10.1186/s13018-021-02702-0

**Published:** 2021-09-15

**Authors:** Yang Tan, Lingxiao Jiang, Hankun Liu, Zhengqi Pan, Hua Wang, Liaobin Chen

**Affiliations:** Division of Joint Surgery and Sports Medicine, Department of Orthopedic Surgery, Zhongnan Hospital of Wuhan University, Wuhan, 430071 Hubei Province China

**Keywords:** Hypoalbuminemia, Hip arthroplasty, Risk factors, Postoperative complications

## Abstract

**Objectives:**

To explore the risk factors of preoperative hypoalbuminemia and its’ effects on complications in the elderly with primary hip arthroplasty.

**Methods:**

A total of 211 elderly inpatients who underwent hip arthroplasty were collected. All patients were divided into the control group (preoperative serum albumin ≥35 g/L) and case group (preoperative serum albumin <35 g/L). The risk factors of preoperative hypoalbuminemia and the postoperative complications were analyzed.

**Results:**

Compared to controls, hypoalbuminemia patients were older (*P* = 0.026), had lower BMI (*P* = 0.045), higher cardiac function score (*P* < 0.0001), higher ASA scores (*P* = 0.023), and longer hospital stay (*P* < 0.001). The intraoperative albumin loss in the case group was significantly higher than that of in control group (*P* < 0.001), but there was no significant difference in operation time and intraoperative blood loss between the two groups (*P* >0.05). Compared to controls, hypoalbuminemia patients had a higher risk for any complication (*P* = 0.014), such as delayed wound healing, pleural effusion, and pneumonia. The risk of postoperative complications increased by 6.9% with every 1 year old is increasing (age > 60). The risk of postoperative complications in the case group was 1.89 times higher than that in the control group.

**Conclusion:**

Patients with older age, poor nutritional status, and more than 2 concomitant diseases are more likely to develop preoperative hypoalbuminemia. Preoperative hypoalbuminemia is related to the increased incidence of postoperative complications. Perioperative albumin loss is not only due to perioperative blood loss, but also related to vascular permeability and abnormal albumin metabolism.

## Introduction

Serum albumin is the most abundant protein in human plasma, which plays an important role in maintaining normal physiological function [1]. Clinically, hypoalbuminemia was defined as a serum albumin <35 g/L [2]. It is very common in a clinic, and it often occurs in elderly patients with chronic diseases such as hypertension, diabetes, and malnutrition. With the increasing “aging” of the society, more and more elderly patients need hip arthroplasty surgery. However, the elderly has the low compensatory ability, more concomitant diseases, and insufficient ability to tolerate hypoalbuminemia. Perioperative hypoalbuminemia has a great impact on the elderly, and the risk of postoperative complications such as wound infection, pneumonia, and limb swelling also increases [3].

At present, many studies have proved that hypoalbuminemia is related to many postoperative complications and adverse consequences, such as wound infection, pneumonia, and cardiac arrest [4–6]. However, most of these studies focus on preoperative malnutrition and set preoperative hypoalbuminemia as one of the indicators. In addition, the samples of some studies include not only patients with an initial hip replacement, but also patients with knee arthroplasty and joint revisions. Therefore, there is no precise conclusion on the incidence of preoperative hypoalbuminemia and postoperative complications in the elderly with primary hip arthroplasty. So, the aim of this study is to investigate the risk factors of perioperative hypoalbuminemia in hip arthroplasty and the relationship between preoperative albumin level and postoperative complications in the elderly. All the elder patients were divided into the control group (preoperative serum albumin ≥35 g/L) and case group (preoperative serum albumin <35 g/L) so that the perioperative conditions and postoperative complications of the two groups were observed and analyzed retrospectively. The results of the study could provide a basis for improving the prognosis of patients after hip arthroplasty.

## Material and methods

### Material

The data of 211 patients who underwent primary hip arthroplasty from January 2019 to December 2020 were collected. Among them,118 patients underwent total hip arthroplasty (THA), and 93 cases underwent hemi-hip arthroplasty (HHA). There were 70 males, 141 women, aged from 60 to 95 years old, the average age was 74.21 ± 8.46 years old, and the average BMI was 22.50 ± 2.40 kg/m^2^(Table 1). There were 115 cases of a femoral neck fracture, 22 cases of the intertrochanteric femur, 32 cases of osteoarthritis, 32 cases of osteonecrosis of the femoral head, and 10 cases of congenital dysplasia of the hip joint. The inclusion criteria are as follows: (1) primary hip arthroplasty, (2) patients whose age was ≥ 60 years old, and (3) patients with complete case data and examination results. The exclusion criteria are as follows: (1) revision hip arthroplasty, (2) patients less than 60 years old, (3) patients who underwent bilateral hip arthroplasty at the same time, (4) patients with a history of operation or infection of the hip joint, and (5) patients with hematological diseases, blood coagulation disorders, known malignant tumors, or infections.

**Table 1:** Comparison of general data between two groups of patients

Factors	Control group	Case group	*χ*^2^ or *t* value	95%CI	*p value*
Age (years),‾*x*±s	73.20±8.04	75.88±8.92	2.249	0.331–5.022	0.026
Sex (*n*, %)			0.737	0.429–1.392	0.391
Man	40 (30.53%)	29 (36.25%)			
Woman	91 (69.47%)	51 (63.75%)			
BMI (kg/m^2^)	22.76±2.57	22.08±2.03	2.021	-1.351– -0.017	0.045
Hospital stay (days)	12.46±3.16	14.99±5.72	4.132	1.323–3.736	<0.001

### Methods

According to the preoperative serum albumin level, all patients were divided into the control group (preoperative serum albumin ≥ 35g/L) and case group (preoperative serum albumin < 35g/L): collecting the case information and clinical data of all patients and recording the perioperative laboratory data of all patients (serum albumin, hemoglobin, C-reactive protein, etc.), preoperative health status and intraoperative blood loss, operation time, and intraoperative blood loss as well as the occurrence of postoperative complications.

Patients in both groups were treated with artificial hip arthroplasty by the lateral approach. According to the patient’s age, femoral neck fracture, daily activity, and other indicators, the chief surgeon decided to perform THA or HHA. In this study, all cases were treated with uncemented joint replacement. For the patients with THA, the drainage tube was indwelled after the operation and removed within 48 h after operation. The patients with HHA had no drainage tube after the operation. Guide and assist the patient to walk 1–2 days after the operation. All patients were routinely given antibiotics to prevent infection for 24 h after arthroplasty, and oral anticoagulants continued for 4 weeks after 1 week of subcutaneous injection of anticoagulants (if unexpected, stop in time). Patients were given intravenous albumin supplementation if their serum albumin was less than 30g/L at 3 days after the operation.

### Statistical analysis

Frequency (percentage) was calculated for qualitative data, and the mean ± standard deviation was calculated for quantitative data. An independent t test or Wilcoxon rank-sum test was used to examine the difference in continuous variables between groups, whereas the chi-square test or Fisher’s exact test was used to compare the difference in categorical variables. A 2-tailed *P* value < 0.05 was considered statistically significant. All statistical analyses were performed with SPSS, version 22.0, software (SPSS Inc., Chicago, IL).

## Results

### Demographic and preoperative data

A total of 211 hip arthroplasty patients were included in the study. They were divided into control group (*n* = 131) and case group (*n* = 80). Compared with the control group, the patients in the case group were older (75.88 ± 8.92 vs 73.20 ± 8.04 years, *P* = 0.026), BMI was lower (22.08 ± 2.03 vs 22.76 ±2.57 kg/m^2^, *P* = 0.045), and hospital stay was longer (14.99 ± 5.72 vs 12.46 ± 3.16 days, *P* < 0.001), ASA score and cardiac function score in case group were higher than that of in control group (*P* < 0.05). There was no significant difference in diagnosis (*P* = 0.643), mode of operation (*P* = 0.619), and mode of anesthesia (*P* = 0.937) between the two groups (Tables 1 and 2).

**Table 2:** Preoperative data of two groups of patients

Factors	Control group	Case group	*χ*^2^ value	*p value*
Diagnosis (*n*, %)				
Femoral neck fracture	75 (57.3%)	40 (50.0%)	3.372	0.643
Intertrochanteric fracture of femur	11 (8.4%)	11 (13.8%)		
Osteonecrosis of femoral head	21 (16.0%)	11 (13.8%)		
Osteoarthritis of hip	17 (13.0%)	15 (18.7%)		
Congenital dysplasia of hip joint	7 (5.3%)	3 (3.8%)		
ASA score (*n*, %)				
1	7 (5.3%)	0 (0.0%)	7.517	0.023
2	89 (67.9%)	48 (60.0%)		
3	35 (26.7%)	32 (40.0%)		
Cardiac function (*n*, %)				
1	45 (34.4%)	1 (1.3%)	31.92	<0.001
2	76 (58.0%)	70 (87.5%)		
3	10 (7.6%)	9 (11.2%)		
Mode of operation (*n*,%)				
THA	75 (57.3%)	43 (53.8%)	0.247	0.619
HHA	56 (42.7%)	37 (46.2%)		
Anesthetic mode (*n*, %)				
General anesthesia	94 (71.8%)	57 (71.2%)	0.006	0.937
CSEA	37 (28.2%)	23 (28.8%)		

Among the concomitant diseases before the operation, the proportion of cardiovascular system, diabetes, bedsores, abnormal liver, and kidney function and more than two kinds of concomitant diseases in the case group was significantly higher than that in the control group (*P* < 0.05). There was no significant difference in the respiratory and digestive system diseases between the two groups (*P* > 0.05) (Table 3).

**Table 3:** Concomitant disease in two groups of patients

Types of concomitant diseases (*n*, %)	Control group	Case group	*χ*^2^ value	Odds ratio (95%CI)	*p value*
Respiratory system	8 (6.1%)	7 (8.8%)	0.526	0.678 (0.236–1.948)	0.469
Digestive system	4 (3.1%)	4 (5.0%)	0.516	0.598 (0.145–2.463)	0.473
Bedsore	1 (0.8%)	4 (5.0%)	3.853	0.146 (0.016–1.332)	0.050
Diabetes	11 (8.4%)	15 (18.8%)	4.928	0.397 (0.182–0.891)	0.026
Cardiovascular system	52 (39.7%)	45 (56.3%)	5.481	0.512 (0.291–0.900)	0.019
Abnormal renal function	14 (10.7%)	21 (26.3%)	8.994	0.331 (0.157–0.696)	0.003
Abnormal liver function	10 (7.6%)	19 (23.8%)	10.880	0.265 (0.116–0.606)	0.001
More than 2 concomitant diseases	22 (16.8%)	25 (31.3%)	5.995	0.444 (0.230–0.858)	0.014
Without concomitant disease	27 (20.6%)	7 (8.8%)	5.169	2.707 (1.148–6.977)	0.023

### Intraoperative condition and perioperative albumin changes

**Fig. 1:**
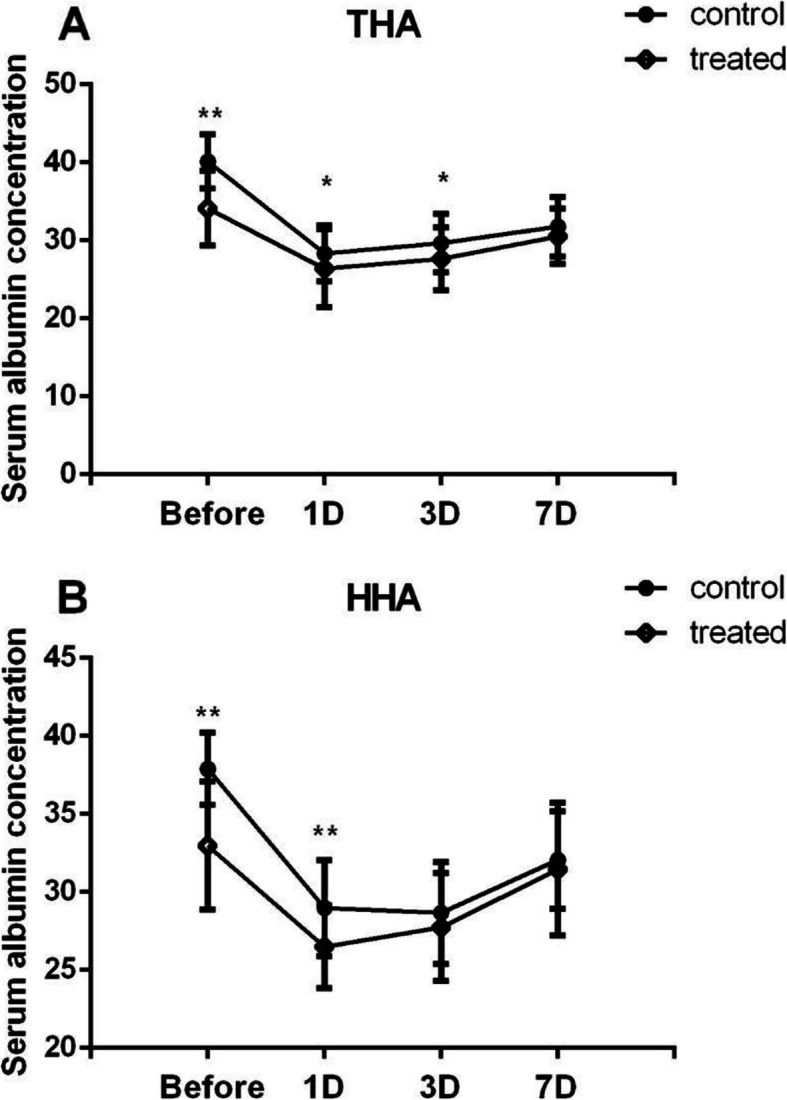
The changes of serum albumin in the two groups during perioperative period. **A** Perioperative albumin level in patients with THA. **B** Perioperative albumin level in patients with HHA. Data presented as mean ± SD, ^*^P<0.05, and ^**^P<0.01

**Fig. 2:**
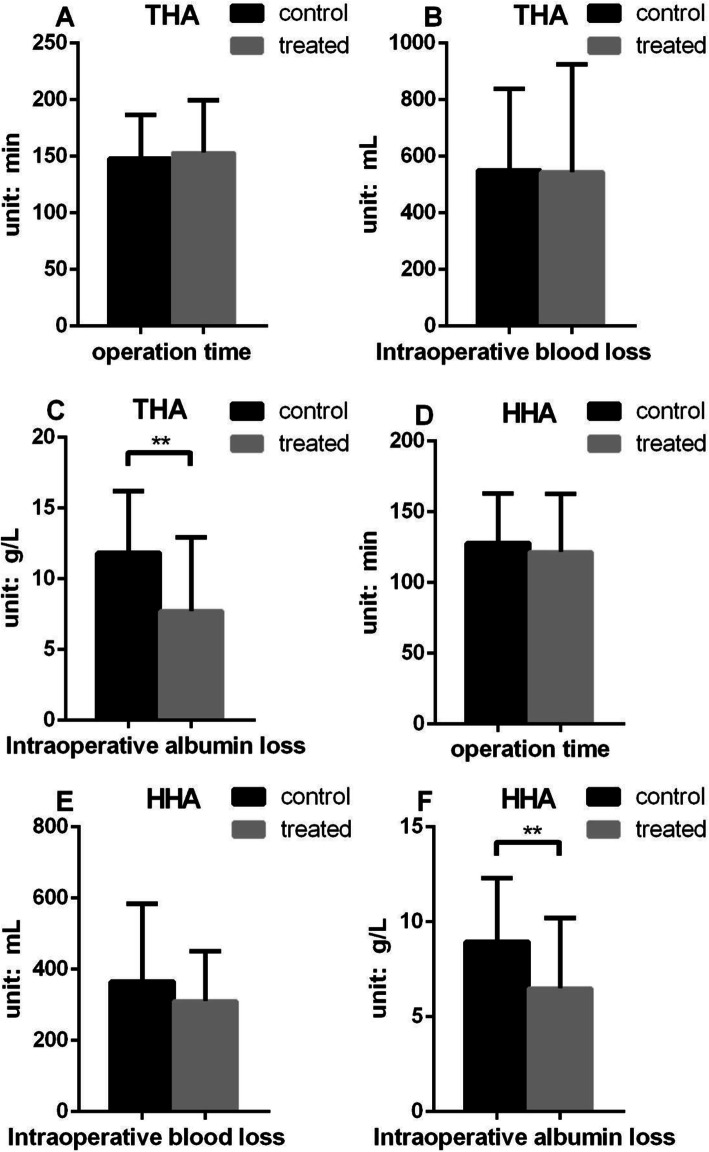
The intraoperative condition of the patients in the two groups. **A**–**C** Intraoperative condition of patients with THA; D. E. F. Intraoperative condition of patients with HHA. (intraoperative blood/albumin loss = preoperative blood/albumin level – 1 day postoperative blood/albumin level). Data presented as mean ± SD, ^*^P<0.05, and ^**^P<0.01

All patients underwent THA or HHA surgery. Because the two surgical methods may affect the operation time, intraoperative blood loss, and other related factors, the control group was divided into the THA control group (*n* = 75) and HHA control group (*n* = 56), and the case group was divided into the THA case group (*n* = 43) and HHA case group (*n* = 37). Compared with the THA control group, the average serum albumin level before the operation, 1 day and 3 days after the operation, was lower in the THA case group (*P* < 0.05), but there was no significant difference on the 7th day after the operation (*P* > 0.05). Compared with the HHA control group, the average level of serum albumin before and 1 day after the operation was lower in the HHA case group (*P* < 0.05), but no significant difference at 3 and 7 days after operation (*P* > 0.05) (Fig. 1). The intraoperative albumin loss in the control group was significantly higher than that of in case group (*P* < 0.001), but there was no significant difference in operation time and intraoperative blood loss between the two groups (*P*>0.05) (Fig. 2).

### Postoperative complication

Compared with the control group, the incidence of total postoperative complications in the case group was higher. (OR = 0.492; 95% CI, 0.278–0.868; *P* = 0.014). Among them, the risk of delayed wound healing, pleural effusion, and pneumonia in the case group was significantly higher than that of in control group (*P* < 0.05). There was no significant difference in the incidence of deep venous thrombosis, dyspepsia, constipation, and electrolyte disturbance between the two groups (*P* > 0.05) (Table 4).

**Table 4:** Occurrence of postoperative complications in two groups of patients

Complication	Control group (*n*, %)	Case group (*n*, %)	*χ*^2^*value*	Odds ratio (95%CI)	*p value*
Total complications	59 (45.0%)	50 (62.5%)	6.065	0.492 (0.278–0.868)	0.014
Delayed wound healing	14 (10.7%)	20 (25.0%)	7.528	0.359 (0.169–0.761)	0.006
Pleural effusion	1 (0.8%)	5 (6.3%)	5.412	0.115 (0.013–1.007)	0.020
pneumonia	1 (0.8%)	6 (7.5%)	7.028	0.095 (0.011–0.804)	0.008
Deep venous thrombosis of lower extremities	7 (5.3%)	3 (3.8%)	0.279	1.449 (0.364–5.773)	0.597
Dyspepsia	5 (3.8%)	6 (7.5%)	1.364	0.489 (0.144–1.660)	0.243
Constipation	17 (13.0%)	17 (21.3%)	2.515	0.553 (0.264–1.158)	0.113
Electrolyte disorder	18 (13.7%)	8 (10.0%)	0.643	1.434 (0.592–3.470)	0.423
Death	0 (0%)	1 (1.3%)	1.645	0 (0–5.496)	0.200

In order to explore the influence of sex, age, BMI, fracture, previous medical history, mode of operation, preoperative hypoalbuminemia, and other factors on the occurrence of postoperative complications, the related factors were assigned (Table 5), and the univariate analysis of related factors was made (Table 6). It is suggested that sex, age, mode of operation, and preoperative hypoalbuminemia have an influence on the occurrence of postoperative complications (*P*<0.1). Binary logistic regression analysis was performed on the univariate factors related to sex, age, mode of operation, and preoperative hypoalbuminemia. The results showed that the Hosmer-Lemeshow inspection *χ*^2^=2.899, *P*=0.941 (*P* > 0.05). It is suggested that there is no statistically significant difference between the predicted value of the model and the actual observed value, and the prediction model has good calibration ability. Sex and mode of operation were not independent influencing factors of postoperative complications (*P* > 0.05), but age and preoperative hypoalbuminemia could significantly affect the occurrence of postoperative complications (*P* < 0.05). The risk of postoperative complications increased by 6.9% with every 1 year old is increasing (age >60). And the risk of postoperative complications in patients with preoperative hypoalbuminemia (serum albumin < 35g/L) was 1.89 times higher than that in patients with normal preoperative albumin(preoperative serum albumin ≥ 35g/L) (Table 7).

**Table 5:** Variables and assignment of risk factors for postoperative complications

Factors	Assignment
Sex	0= male, 1= female
Fracture	0= no, 1= yes
Previous medical history	0= no, 1= yes
Mode of operation	0= HHA, 1= THA
Preoperative albumin level (g/L)	0=≥35, 1=<35
Postoperative complication	0=no, 1= yes

**Table 6:** Single-factor comparison of exposure factors with or without postoperative complications

Factors	Postoperative complications (*n*=109)	No postoperative complications (*n*=102)	*t value*	*p value*	95%CI
Sex, male (%)	30 (27.52)	39 (38.24)	1.166	0.098	−0.020–0.234
Age (years), *x*±s	76.45±8.54	71.82±7.74	4.114	<0.001	−6.843– -2.409
BMI(kg/m^2^), *x*±s	22.47±2.51	22.53±2.29	0.1774	0.859	−0.595–0.712
Fracture, yes (%)	74 (67.89)	61 (59.80)	1.221	0.223	−0.211–0.050
Previous medical history, yes (%)	88 (80.73)	83 (81.37)	0.1177	0.906	−0.101–0.113
Mode of operation, THA (%)	55 (50.46)	63 (61.76)	1.656	0.099	−0.022–0.248
Preoperative albumin level, <35 (g/L),‾*x*±s	50 (45.87)	30 (29.41)	2.487	0.014	−0.295– -0.034

**Table 7:** Binary logic regression analysis of related factors

Factors	*B*	S.E.	Wald	*p value*	Odds ratio	95%CI
Sex	−0.598	0.324	3.399	0.065	0.550	0.291–1.038
Age	0.066	0.019	11.988	0.001	1.069	1.029–1.110
Mode of operation	−0.004	0.317	<0.001	0.989	0.996	0.535–1.853
Preoperative albumin level	0.636	0.305	4.340	0.037	1.889	1.038–3.436

## Discussion

In this study, according to the set inclusion and exclusion criteria, all eligible patients from January 2019 to December 2020 were included, including 131 in the control group and 80 in the case group. Although the number of people in the control group is more than that in the case group, the situation is natural and closer to the real world than the equal number of people in the control group and the case group.

### Main discovery

This study found that patients with more preoperative concomitant diseases were more likely to develop hypoalbuminemia. According to the results of this study, the proportion of patients with cardiovascular disease, diabetes mellitus, bedsore, abnormal liver and kidney function, or more than 2 concomitant diseases before the operation was higher in the case group (*P* < 0.05). Hypertension is one of the most common cardiovascular diseases in the world. It has been found that the pathogenesis of hypertension is closely related to the damage of vascular endothelial function [7]. Microvascular permeability is increased in patients with hypertension. When complicated with trauma or surgery, a large number of inflammatory cytokines are released, which aggravates the injury of capillary endothelial cells, causes vascular leakage, results in hypoalbuminemia [8]. In patients with diabetes, their insulin receptor is inhibited under stress, the oxidative metabolism of glucose is abnormal, and the negative nitrogen balance is more stubborn and obvious [9]. Abnormal liver and kidney function will reduce its protein synthesis ability and plasma protein synthesis, such as hypoproteinemia caused by cirrhosis.

Patients with hypoalbuminemia were more likely to have postoperative complications. Compared with the control group, patients with hypoalbuminemia had a higher incidence of postoperative complications, especially delayed wound healing, pleural effusion, and pneumonia. And there was no significant difference in deep venous thrombosis, dyspepsia, constipation, and electrolyte disturbance of lower extremities. Meanwhile, we used binary logistic regression analysis to analyze the correlation between sex, age, BMI, fracture, and preoperative albumin level, the result showed that the risk of postoperative complications increased by 6.9% with every 1 year old is increasing (age >60), and the risk of postoperative complications in patients with preoperative hypoalbuminemia (serum albumin < 35g/L) was 1.89 times higher than that in patients with normal preoperative albumin (preoperative serum albumin ≥ 35g/L).

In this study, there was one dead patient who was admitted to our hospital for surgical treatment because of a femoral neck fracture. The patient had a history of hypertension and after hysterectomy for endometrial carcinoma. The causes of death were acute renal failure, acute cerebral infarction, and metabolic acidosis. Although the patient had hypoalbuminemia before the operation, the main cause of death was preoperative abnormal liver and kidney function and basic physical condition, rather than preoperative hypoalbuminemia.

In addition, we found that intraoperative blood loss cannot be used to predict albumin loss separately. The results showed that the amount of postoperative albumin loss in the case group was significantly lower than that in the control group, no matter in the patients who received THA or HHA (*P* < 0.05), but there was no significant difference in operation time and intraoperative blood loss between the two groups (*P* > 0.05). It is suggested that intraoperative blood loss is not the only cause of postoperative albumin loss, but is also related to vascular permeability or abnormal albumin metabolism.

As for the changes of serum albumin during the perioperative period, the results of this study showed that the serum albumin levels of the THA case group before the operation, 1 day and 3 days after the operation was lower than those of the THA control group (*P* < 0.05), but there was no significant difference on the 7 days after the operation (*P* > 0.05). And the albumin level in the HHA case group was lower than that in the HHA control group before operation and 1 day after the operation, but there was no significant difference between 3 days and 7 days after the operation (*P* > 0.05). All patients in this study were given exogenous albumin supplementation if their serum albumin was less than 30g/L at 3 days after the operation. This may be one of the main reasons why there is no significant difference in albumin level between the two groups 7 days after the operation.

### Compared with previous studies

The results of this study are consistent with other findings that have a higher incidence of complications in malnourished patients receiving THA. However, most studies only regard preoperative hypoalbuminemia as one of the influencing factors and do not deeply study the relationship between preoperative hypoalbuminemia and postoperative complications of THA. At the same time, other studies are different from the results of this study in some aspects. Newman et al. [6] found that compared with the control group, hypoalbuminemia patients had an 80% higher risk of any complications, a 113% higher risk of major complications (such as pulmonary embolism, acute renal failure, myocardial infarction, etc.), and a 79% increased risk of minor complications (such as wound infection, blood transfusion, lower extremity deep venous thrombosis), and a 97% increase in reoperation risk. The high incidence of postoperative complications was different in our study. This may be related to factors such as the ratio of fracture patients and the different preoperative concomitant diseases in the two samples.

In addition, preoperative hypoalbuminemia also affects the incidence of complications after other joint replacements. Blevins et al. [10] found that preoperative hypoalbuminemia was a high-risk factor for PJI and showed good sensitivity and specificity in predicting PJI. Kamath et al. [4] evaluated patients treated with TKA and found that patients with hypoalbuminemia had higher incidences of deep surgical site infection, pneumonia, urinary tract infection, and sepsis than those with normal albumin levels. Patients with hypoalbuminemia had a higher risk of death and coma than patients with hypoalbuminemia who required unplanned tracheal intubation, intraoperative or postoperative blood transfusion, retention of the ventilator for more than 48 h, and coma. Any complications and infections (systemic and traumatic) are also more common. However, in our study, although there was one death in preoperative hypoalbuminemia, the cause of death was mainly due to poor basic conditions and abnormal liver and kidney function, rather than hypoalbuminemia. These studies have shown that preoperative hypoalbuminemia can significantly affect the incidence of complications after joint replacement.

### Limitations

We admit that there are still some limitations in this study. The main cases included in this study were traumatic patients with femoral neck fracture and intertrochanteric fracture (64.9%), while fewer patients underwent elective surgery such as osteonecrosis of the femoral head and osteoarthritis of the hip joint (35.1%). Some studies have shown that compared with elective patients, trauma patients have older age, longer hospital stay, more intraoperative complications, and lower preoperative serum albumin levels [11]. Furthermore, only patients over 60 years old were included in the study. Compared with young and middle-aged patients, elderly patients were more likely to be complicated with underlying diseases such as cardiovascular disease, abnormal liver, and kidney function, and these basic diseases may cause patients’ albumin levels to be lower than normal. And because of the retrospective single-center nature of this study, the extensibility of this study is limited.

## Conclusion

In this study, we found that hypoalbuminemia patients who needed primary hip replacement were more elderly, poor nutritional status, cardiovascular system, diabetes, bedsores, liver and kidney dysfunction, and other concomitant diseases. Patients with hypoalbuminemia have a longer hospital stay and a higher incidence of postoperative complications such as delayed wound healing and pneumonia. At the same time, the cause of postoperative hypoalbuminemia is not only blood loss during the perioperative period, but is also related to abnormal vascular permeability and albumin metabolism.

We innovative found that the risk of postoperative complications increased by 6.9% with every 1 year old is increasing (age >60). And the risk of postoperative complications in patients with preoperative hypoalbuminemia (serum albumin < 35g/L) was 1.89 times higher than that in patients with normal preoperative albumin(preoperative serum albumin ≥ 35g/L).

Therefore, for patients with hypoalbuminemia who need a primary hip replacement, active treatment of concomitant diseases before operation and adequate albumin supplement in the perioperative period are helpful to reduce the risk of postoperative complications.

## Data Availability

All data are included in the manuscript.

## Abbreviations

ASA: American Society of Anesthesiologists classification of physical condition
BMI: Body mass index
CSEA: Combined Spinal Epidural Anesthesia
HHA: Hemi-hip arthroplasty
THA: Total hip arthroplasty
